# Determinants of viral suppression among adolescents on antiretroviral treatment in Ehlanzeni district, South Africa: a cross-sectional analysis

**DOI:** 10.1186/s12981-021-00391-7

**Published:** 2021-10-09

**Authors:** Emeka F. Okonji, Brian van Wyk, Ferdinand C. Mukumbang, Gail D. Hughes

**Affiliations:** 1 School of Public Health, University of the Western Cape, P Bag X17, Bellville, 7535 South Africa; 2 Department of Global Health, University of Washington, Seattle, WA USA; 3 Medical Biosciences Department, University of the Western Cape, Bellville, South Africa

**Keywords:** Adolescents, Viral suppression, Interventions, HIV and AIDS, Adherence and retention

## Abstract

**Background:**

Achieving undetectable viral load is crucial for the reduction of HIV transmissions, AIDS-related illnesses and death. Adolescents (10 to19 years) living with HIV (ALHIV) on antiretroviral treatment (ART) have worse treatment adherence and lower viral suppression rates compared to adults. We report on the clinical factors associated with viral suppression among ALHIV in the Ehlanzeni district, Mpumalanga in South Africa.

**Methods:**

A cross-sectional analysis was conducted with 9386 ALHIV, aged 10 to 19 years, who were enrolled in 136 ART clinics in the Ehlanzeni district. Clinical and immunological data were obtained from electronic medical records (Tier.net). ALHIV were categorised as having achieved viral suppression if their latest viral load count was < 1000 ribonucleic acid (RNA) copies/mL. Using a backward stepwise approach, a multivariate logistic regression analysis was performed to identify factors independently associated with viral suppression.

**Results:**

The mean age of the participants was 14.75 years (SD = 2.9), and 55.43% were female. Mean duration on ART was 72.26 (SD = 42.3) months. Of the 9386 adolescents with viral load results recorded, 74% had achieved viral suppression. After adjusting for other covariates, the likelihood of achieving viral suppression remained significantly higher among ALHIV who were: female (AOR = 1.21, 95% CI 1.05–1.39), and had most recent CD4 count > 200 (AOR = 2.53, 95% CI 2.06–3.11). Furthermore, the likelihood of having viral suppression was lower among adolescents with CD4 count > 200 at baseline (AOR = 0.73, 95% CI 0.61–0.87), and who were switched to second line regimen (AOR = 0.41, 95% CI 0.34–0.49).

**Conclusions:**

Viral suppression amongst ALHIV at 74% is considerably lower than the WHO target of 95%. Of particular concern for intervention is the lower rates of viral suppression amongst male ALHIV. Greater emphasis should be placed to early enrolment of ALHIV on ART and keeping them engaged in care (beyond 6 months). Furthermore, improved and regular viral load monitoring will help to adequately identify and manage ALHIV with unsuppressed viral load and subsequently switching to second line treatment.

**Supplementary Information:**

The online version contains supplementary material available at 10.1186/s12981-021-00391-7.

## Introduction

In 2018, UNAIDS estimated that 1.6 million young people aged 10 to 24 years were living with HIV [1, 2]. Therefore, young people living with HIV constitute a growing and key sub-population of people living with HIV globally. The increase in HIV prevalence amongst adolescents (10 to 19 years) is the result of the generation of children infected with HIV perinatally who are surviving into adolescence because of improved access to antiretroviral treatment (ART), and increased HIV incidence as a result of risky (sexual) behaviour in this age group [3, 4]. Despite tremendous gains in the reduction of over-all AIDS-related deaths to the tune of 43%, AIDS-related deaths amongst adolescents in Eastern and Southern Africa have increased in the last decade [5]. This is mainly because adolescents struggle to initiate and remain engaged on antiretroviral treatment (ART) [6].

Monitoring the level of detectable viral load and achieving undetectable viral loads are crucial in the reduction of HIV transmission, earlier detection of treatment failure, and timely switching to second-line ART [7–10]. The aim of ART is to suppress the replication of the HIV thereby protecting people living with HIV from AIDS-related illnesses and death and preventing further transmission to others [3, 11]. Conversely, adolescents who have poor ART adherence are at greater risk of morbidity, mortality, treatment failure, the development of drug resistant forms of HIV, viral progression, opportunistic infections, and transmission to babies and sexual partners [8, 12–16].

Compared to adult populations living with HIV, adolescents living with HIV (ALHIV) have a higher likelihood of suboptimal adherence, viral load progression, lost to follow-up, morbidity and mortality [11, 17, 18]. This is because adolescence is accompanied by rapid physical, psychological and physiological changes, which influence health‐related behaviour [19]. Therefore, monitoring the treatment outcomes for ALHIV is crucial as they become aware of their HIV status and have to navigate health care facilities and self-manage their medication adherence and retention in ART care [20]. Regrettably, there is limited information on viral outcomes of adolescents in sub-Saharan Africa where the biggest burden of deaths is experienced, because these results are masked by routine reporting for children under 14 years and adults from 15 years up only [17, 19].

The aim of this study is to investigate the predictors of viral load suppression among HIV-positive adolescents (10 to 19 years) receiving ART in the Ehlanzeni district of South Africa. Improved reporting of virological outcomes from a wider range of settings is required to support efforts to improve HIV care and treatment for adolescents [19].

## Setting

Ehlanzeni District municipality is in Mpumalanga province, located in the Northern Eastern part covering the whole Southern part of the Kruger National Park. The district is surrounded by Mozambique in the East and Swaziland in the South. The district comprises of four sub-districts namely: Bushbuckridge, City of Mbombela, Nkomazi, and Thaba Chweu.

According to the 2017 South African National HIV prevalence, incidence, behaviour and communication survey, among people living with HIV in South Africa, Mpumalanga province has the second highest HIV prevalence with an estimated prevalence rate of 17.3%, and the lowest in viral load suppression (82.9%) [21, 22]. Furthermore, the Ehlanzeni district compared to other districts has high rate of people living with HIV who know their HIV status (1^st^ 95), 90.6%. On the contrary, ART coverage (2^nd^ 95) remains low at 67.2%, and lowest rate of viral load suppression at 65.3% [21].

## Methods

### Study design and participants

A cross-sectional analysis of routine data on ALHIV, aged 10 to 19 years, who were registered to receive ART from 136 clinics in the Ehlanzeni district between September 2002 and October 2019 was conducted. On October 31, 2019, we extracted anonymized individual patient data (sociodemographic variables), clinical data and treatment outcomes (viral load result) of ALHIV who have been on ART for at least 6 months from electronic medical records (TIER.net) as part of a larger study assessing the effects of psychosocial support on adherence, treatment outcomes (viral suppression) and retention in care amongst adolescents on ART. The Tier.net electronic record only records the last viral load test done; CD4 count at baseline and most recent CD4 count.

ALHIV who have been on ART for less than 6 months, without viral load done, who have died, lost to follow-up, and transferred/moved out were excluded from the analysis. Only ALHIV with viral load done after 6 months of ART initiation were included in the analysis (see Additional file 1: Appendix S1 for inclusion and exclusion criteria).

The primary outcome, viral suppression, was defined according to the South African National Department of Health as patients achieving a viral load < 1000 RNA copies/mL [23]. In South Africa, the first viral load test is done 6 months after initiation on ART. The predictor variables such as age, gender, method of entry into ART programme at the health facility, pregnant on ART, age at ART initiation, duration on ART, initiated on Isoniazid Preventive Therapy (IPT), TB history, CD4 count at baseline and last ART visit (most recent CD4 count), WHO stage at initiation, and initiated on ART same day as HIV diagnosis were employed in a bivariate and multivariate analysis to determine factors influencing viral suppression.

### Analysis

Data were extracted from the Tier.Net medical electronic record in Excel, and imported to STATA statistical software version 16.0 (STATA Corporation, College Station, Texas, USA) for analyses. Information on clinical stationery was reviewed against information on Tier.net. Patient clinical records identified as incomplete or not correctly captured on Tier.net was retrieved and subsequently updated on Tier.Net.

Descriptive statistics were used to characterize the demographic and clinical variables (at baseline and/or  six months after ART initiation). Comparisons between viral suppression and clinical parameters among adolescent living with HIV were achieved using chi-square tests for proportions (replaced by Fisher’s exact test for sparse data), and bivariate logistics regression analysis to examine associations.

Furthermore, a multivariate logistic regression model was used to estimate factors associated with viral suppression adjusting for potential confounders using the following variables: age, gender, age at ART initiation, duration on ART, CD4 cell count at baseline and most recent CD4 cell count, WHO stage at initiation, and initiated on ART same day as HIV diagnosis. The multivariate logistic regression model employed a backward stepwise analysis. In the backward selection model, we included all candidate variables in the model with *p* < 0.15 in selecting the final model [24]. At each step, the variable that is the least significant is removed. This process continued until no non-significant variables remained. The significance level was set at 95% at which variables can be removed from the model. Analyses were conducted among all patients with viral load test done after 6 months of ART initiation.

### Ethics approval

Ethics clearance was obtained from the University of the Western Cape Biomedical Research Ethics committee (BM19/1/8) and informed consent to use the Tier.Net medical electronic dataset was obtained from the National Health Research Ethics committee (MP_202102_006). We adhered to the 1964 declaration of Helsinki guidelines. According to the declaration, research that involves human subjects amongst others must keep with the following (1) strive to protect life, health, privacy, and the dignity of the research participants, (2) employ greater care to protect the participants from harm and (3) conduct the research because the importance of the research purpose, outweighs the risk that might be attributed to the study either at present or in the future (18th WMA General Assembly, Helsinki, 2001).

Data extraction excluded adolescent’s unique identifiers such as name, surname, patient folder number and identity number.

## Results

Table 1 shows the demographic characteristics of ALHIV enrolled in the ART programme in 136 facilities in Ehlanzeni district South Africa. This study included 9386 adolescents (aged 10 to 19 years) with mean age 14.75 years (SD = 2.9); of whom 55.43% were female. Of the 9386 ALHIV with viral load results recorded, 74.31% had achieved viral suppression. Compared to ALHIV in the age group (15 to 19 years), ALHIV (10 to 14 years) are more likely to have viral load below 1000 RNA copies/mL (p < 0.001).

**Table 1:** Viral load suppression by demographic and clinical characteristics of adolescents 10 to 19 years living with HIV in Ehlanzeni district, South Africa (N = 9386)

	Total	Viral load suppression		
	9386	Yes n (%) 6975 (74.31)	No n (%) 2411 (25.69)	*p*-value
Current age (in years)				0.000
10 to 14	4506 (48.01)	3455 (76.41)	1063 (23.59)	
15 to 19	4880 (51.99)	3603 (72.38)	1348 (27.62)	
Gender				0.000
Female	5158 (54.95)	3946 (76.50)	1212 (23.50)	
Male	4253 (44.57)	3029 (71.64)	1199 (28.36)	
Pregnant at ART start (N = 5160)				0.001
No	4729 (91.65)	3590 (75.91)	1139 (24.09)	
Yes	431 (8.35)	357 (82.83)	74 (17.17)	
Age at ART start (in years)				0.000
0 to 9	5512 (58.73)	4116 (74.67)	1396 (25.33)	
10 to 14	2548 (27.15)	1786 (70.09)	762 (29.91)	
15 to 19	1326 (14.13)	1073 (80.92)	253 (19.08)	
Duration on ART (in months)				0.002
6 to 11 months	459 (4.89)	364 (79.30)	95 (20.70)	
12 to 17 months	450 (4.79)	332 (73.78)	118 (26.22)	
18 to 24 months	458 (4.88)	366 (79.91)	92 (20.09)	
25 months +	8019 (85.44)	5913 (73.74)	2106 (26.26)	
Initiated on Isoniazid Preventative Therapy (IPT)				0.002
Yes	2111 (22.49)	1623 (76.88)	488 (23.12)	
No	7,275 (77.51)	5352 (73.57)	1923 (26.43)	
History of TB				0.009
Yes	78 (0.87%)	48 (61.54%)	30 (38.46%)	
No	8934 (99.13%)	6659 (74.54%)	2275 (25.46%)	
CD4 count at last ART visit (n = 7999)				
< 200	1071 (13.39%)	608 (56.77%)	463 (43.23%)	0.000
> 200	6928 (86.61%)	5284 (76.27%)	1644 (23.73%)	
CD4 count at baseline				0.006
CD4 < 200	1676 (28.16%)	1200 (71.60%)	476 (28.40%)	
CD4 > 200	4276 (71.84%)	3210 (75.07%)	1066 (24.93%)	
WHO stage at initiation				0.000
1	4067 (52.72%)	3106 (76.37%)	961 (23.63%)	
2	1873 (24.28%)	1363 (72.77%)	510 (27.23%)	
3	1527 (19.79%)	1088 (71.24%)	439 (28.75%)	
4	248 (3.21%)	176 (70.97%)	72 (29.03%)	
Initiated same day				0.145
Yes	808 (61.59%)	631 (78.09%)	177 (21.91%)	
No	504 (38.41%)	376 (74.60%)	128 (25.40%)	
Initiated on second line ART				
Yes	1550 (16.51%)	888 (57.29%)	662 (42.71%)	0.000
No	7836 (83.49%)	6087 (77.68%)	1749 (22.32%)	

In terms of gender comparison, females are more likely to have viral load below 1000 RNA copies/mL (p = 0.000). Most (67.88%) of the ALHIV attended the ART programme in the clinic they were initiated as new patients as opposed to transferred in from another clinic. Amongst the female ALHIV, 8.35% were reported pregnant at the time of enrolling into ART.

## Demographic and clinical history

Table 1 shows the clinical history of ALHIV enrolled in the ART programme in Ehlanzeni district South Africa. The mean age at which the adolescents started ART was 8.7 years (SD = 4.8); and 85.44% were on ART for more than 25 months. ALHIV on ART for six to eleven months were more likely to attain viral suppression compared to adolescents on ART for 12 to 17 months and 25 months and above (*p* = 0.002).

Only 22.49% (n = 2111) were started on Isoniazid preventative therapy (IPT) after ART initiation. Only 2.6% completed TB preventive therapy*.* ALHIV who were started on IPT after ART initiation were more likely to attain viral suppression compared to those who did not (*p* = 0.002).

Regarding history of TB, 0.87% had a TB and HIV comorbidity. ALHIV with a history of TB were less likely to have viral load < 1000 RNA copies/mL (*p* = 0.009).

Compared to ALHIV with CD4 count > 200, ALHIV with CD4 count < 200 were less likely to have viral load < 1000 RNA copies/mL (*p* < 0.001). This is true for CD4 count at baseline and CD4 count at last visit or most recent CD4 count.

Slightly more than half (52.72%) of the ALHIV were initiated on ART at WHO stage 1. Compared to ALHIV with WHO stage 1, 2, and 3 at ART initiation, ALHIV with WHO stage 4 were less likely to have viral load < 1000 RNA copies/mL (*p* < 0.001).

Most (61.59%) of the ALHIV were initiated on ART on the same day of HIV diagnosis. However, there was no association between ALHIV’s time of ART initiation with viral load suppression (*p* = 0.145).

Only 16.51% (n = 1550) of ALHIV were switched to second line ART regimen. However, ALHIV who were switched to second line regimen were less likely to attain viral suppression (*p* < 0.001).

### Factors associated with suppression of viral load among adolescent living with HIV (N = 9386)

Table 2 shows the factors associated with viral suppression in a multivariate logistic regression model. After controlling for the effect of other covariates, the likelihood of attaining viral suppression remained significantly higher among female adolescents (AOR = 1.21, 95% CI 1.05–1.39), had most recent CD4 count > 200 (AOR = 2.53, 95% CI 2.06–3.11). ALHIV on second line treatment were less likely to attain viral suppression compared to their reference group (AOR = 0.41, 95% CI 0.34–0.49), followed by ALHIV who had been on ART for 18 to 24 months (AOR = 0.37, 95% CI 0.15–0.93), and ALHIV with CD4 count > 200 at baseline (AOR = 0.73, 95% CI 0.61–0.87).

**Table 2:** Multivariate logistic regression analysis of factors associated with viral suppression among adolescents living with HIV in Ehlanzeni district South Africa (N = 9386)

	Crude OR	95% CI	Adjusted OR	95% CI	
Age					
10 to 14	1*	0.74–0.89	1		
15 to 19	0.81		0.92	0.78–1.09	
Gender					
Male	1*	1.17–1.41	1*		
Female	1.29		1.21	1.05–1.39	
Method into ART at facility					
Transferred in from another facility	1				
New ART patient	0.94	0.85–1.04			
Started IPT					
No	1*		1		
Yes	1.19	1.01–1.25	1.04	0.89–1.23	
History of TB					
No	1*		1		
Yes	0.55	0.35–0.86	0.73	0.37–1.26	
WHO stage					
Stage 1	1*		1		
Stage 2	0.83	0.73–0.94	0.88	0.75–1.04	
Stage 3	0.77	0.67–0.91	0.86	0.72–1.03	
Stage 4	0.76	0.57–1.00	0.85	0.57–1.26	
ART initiation on same day					
No	1				
Yes	1.21	0.93–1.57			
Duration on ART					
6 to 11 months	1*		1*		
12 to 17 months	0.73	0.54–0.99	0.62	0.25–1.55	
18 to 24 months	1.04	0.75–1.43	0.37	0.15–0.93	
25 months +	0.73	0.58- 0.92	0.60	0.22–1.28	
Age at art start					
0 to 9 years	1*		1		
10 to 14 years	0.79	0.72–0.87	0.88	0.74–1.04	
15 to 19 years	1.44	1.24–1.67	1.30	0.97–1.75	
Pregnant during ART start					
No	1*				
Yes	1.53	1.18–1.98			
CD4 count at last visit					
CD4 < 200	1*		1*		
CD4 > 200	2.45	2.14–2.79	2.53	2.06–3.11	
CD4 count at baseline					
CD4 < 200	1*		1*		
CD4 > 200	1.19	1.05–1.36	0.73	0.61–0.87	
Second line					
No	1*		1*		
Yes	0.38	0.34–0.43	0.41	0.34–0.49	

^*^p-value statistically significant at 5%

## Discussion

In this study, we set out to investigate the predictors of viral load suppression among ALHIV receiving ART in the Ehlanzeni district of South Africa. The proportion of ALHIV with viral suppression after six months of ART initiation was relatively high at 74.31% compared to another study conducted in South Africa [20], but falls short of the global target of 95%. Furthermore, our study revealed that being female, and having most recent CD4 count level > 200 were associated with viral suppression. On the other hand, being on ART for more than six months, as well as being on second line treatment are enhancing factors for viral non-suppression.

Evidence on the relationship between gender and viral load suppression is mixed. While one study showed that males were more likely to achieve viral load suppression compared to females [20], another study found that males are more likely to achieve viral non suppression [25]. However, we found that females were more likely to attain viral suppression compared to males. Adherence among males ALHIV is poor compared to females; males have poor treatment seeking behaviours [25] and as such to get males to test for HIV, link and retain them to ART care remains a challenge [26]. The poor treatment outcome reported among males have been attributed to strong gender norms and practices specifically the perception of masculinity inherent within societies in South Africa. In addition, lack of male-friendly services inhibits males from seeking health care services [26–28].

The literature indicates that older age group or adults can achieve viral suppression because they are able to successfully take their ART medication regularly without supervision i.e. possess self-efficacy and self-competency on ART adherence [9]. Interestingly, our study did not show any significant difference between viral suppression among adolescents in the age group 10–14 years and 15–19 years. This is likely because if no support is provided, adolescents (10–19 years) are faced with psychosocial challenges, lack self-efficacy and self-esteem, and are unable to self-manage themselves with regard to medication adherence [29]. However, a retrospective study conducted among adolescents registered in the Cape Metropole ART clinic in South Africa found younger adolescents (10–14 years) were more likely to achieve viral suppression compared to older adolescents (15–19 years). It was reported that the older adolescents face adherence challenges as a result of transitioning from adolescence to adulthood in which they are expected to self-manage themselves with regard to medication adherence [20].

We also found that longer duration (18 to 24 months) on ART was a risk factor for viral non-suppression. This is contrary with evidence that patients on ART for shorter period are more likely to experience virological failure [9]. Our study findings is interesting given that patients who have been on treatment longer have more experience in managing their treatment [9]. Similar findings were reported in a study, which reported that adolescents who had been on ART between 6 and 12 months were more likely to have viral non-suppression (viral load > 400 RNA copies/mL) compared with those who had been on treatment for longer [9].

Immunological treatment failure refers to a CD4 cell count of < 100 cells/µL after 6 months of therapy [9]. According to the WHO guidelines, a decreasing CD4 cell count is considered a proxy marker for treatment failure when viral load monitoring is not available, and should trigger a switch in ART, particularly if the CD4 cell count is < 200 cells/µL [9]. Although the relationship between viral non-suppression and immunological responses, is not always consistent [9], our study found that adolescents with CD4 cell count > 200 cells/µL at last ART visit were more likely to achieve virological suppression. However, the ability of CD4 counts to predict virologic failure is poor [11].

Studies have shown that delayed detection of treatment failure may increase drug toxicity, which in turn lead to the accumulation of drug resistance-associated mutations, hence may result in increased morbidity and mortality [30]. On the contrary, a timely switching to second-line ART after virological failure along with enhanced adherence counselling is a protective factor against viral progression and mortality [10]. However, we included ALHIV who were already initiated on second line ART to determine whether second-line ART would be a successful ‘rescue’ for viral suppression. Our findings showed that adolescents on second-line treatment were less likely to attain viral suppression. It is possible that those on second line regimen could have a history of poor adherence behaviour that continues in spite of being on the ‘rescue’ regimen. There is also evidence that having a history of treatment failure is a risk factor for viral suppression [17]. Furthermore, second-line regimens are more complex than first-line regimens, are often twice daily regimens and have more adverse side effect than first-line regimens hence impacting negatively on adherence. Unfortunately, assessing HIV resistance among HIV naïve patients is challenged by cost and volume of HIV positive patients in South Africa.

### Findings implications

Several implications arise from our findings. First, to achieve the 95% global target by 2030, there is a need to design an intervention (i.e. psychosocial support) aimed at ALHIV to improve self-efficacy and self-competency so that they are able to adhere to their ART medication. Adopting a combination of multiple approaches including psychosocial support intervention may be necessary to improve adherence. For instance, providing social support and focusing on psychosocial needs of adolescents to bolster their self-esteem and self-efficacy will in turn improve their self-management regarding medication adherence and subsequently improve their treatment outcomes.

Timely and accurate identification of virological failure is crucial to avoid misclassification of non-suppression leading to switching to a second-line or third-line which are costly and can lead to viral non suppression. It is recommended that second line regimens especially for ALHIV are simplified and changed to once daily, and less toxic regimens which could improve adherence and in turn lead to viral load suppression. Furthermore, providing enhanced adherence counselling for ALHIV who are males, on second line regimen and being on ART for more than 18 months is very crucial.

### Study limitations

This study has a number of limitations, which should be taken into account when interpreting the findings. First, adolescents who are eligible for viral load assessment but failed to have it done because they were lost to follow-up, died or transferred out, were not included, which could have resulted in overestimating the rate of viral load suppression. Second, as is the case for all cross-sectional studies, it is subject to other risk or confounding factors that may be present but were not measured. For example, household income status, head of household, type of social support and psychosocial well-being. Third, as is the case for routine programme data which is exposed to data quality issues. We were not able to delineate which clients had low CD4 because they were early in treatment and been allowed to have a drop in CD4 before initiation, and those that had been doing well and now had a drop in CD4. Similarly, the type of ART regimen used for first- and second-line treatment were excluded from analysis due to poor data capturing. Finally, the Tier.Net medical electronic record only captures the last viral load test done, as a result, we were unable to measure two consecutive viral load test three months apart for those with VL > 50 RNA copies/mL making it challenging to explain what is going on for those who are not undetectable as recommended by World Health Organisation.

## Conclusion

Viral suppression amongst adolescents at 74% is considerably lower than the WHO target of 95%. Of particular concern for intervention is the lower rates of viral suppression amongst male adolescents. Greater emphasis should be placed to enrol adolescents on ART earlier and keeping them engaged in care (beyond 6 months). Furthermore, improved and regular viral load monitoring will help to adequately identify and manage ALHIV with unsuppressed viral load and subsequently switching to second line treatment.

## Supplementary Information

**Additional file 1: Appendix S1.**

## Data Availability

The datasets generated and/or analysed during the current study are not publicly available due it belonging to the South African National Department of Health, but are available from the corresponding author on reasonable request.

## Abbreviations

ALHIV: Adolescents living with HIV
ART: Antiretroviral treatment
AOR: Adjusted Odds Ratio
HIV: Human immunodeficiency-virus
WHO: World Health Organization
TB: Tuberculosis
YPLHIV: Young people living with HIV
