# Supplementary material for: Determinants of viral suppression among adolescents on antiretroviral treatment in Ehlanzeni district, South Africa: a cross-sectional analysis
Source: AIDS Res Ther. 2021 Oct 9;18:66. doi: 10.1186/s12981-021-00391-7 (PMC8501534; doi:10.1186/s12981-021-00391-7)
Supplement: Supplementary file 1 — Additional file 1: Appendix S1. [file 12981_2021_391_MOESM1_ESM.docx]

Appendix 1: sample inclusion and exclusion criteria

Number of Adolescents on ART for less than 6 months

(n=157)

Number of Adolescents

Died (n=336)

Lost to follow up (n=2,029)

Transferred out/moved out (n=3,050)

Number of Adolescents without viral load done

(n=4,849)

Number of Adolescents with viral load done after 3 months of ART initiation

(N=9,386)

Number of Adolescents started ART between Sept 2002 and Sept 2019

(N=19,827)
